# Supplementary material for: Neural arbitration between social and individual learning systems
Source: eLife. 2020 Aug 11;9:e54051. doi: 10.7554/eLife.54051 (PMC7476763; doi:10.7554/eLife.54051)
Supplement: Supplementary file 1. — MNI coordinates and F-statistic of activations induced by precision-weighted prediction error about individually estimated card color probability (Equation 14). Related to Figure 6—figure supplement 1a. (B) MNI coordinates and F-statistic of activations induced by precision-weighted prediction error about advice validity (Equation 8). Related to Figure 6—figure supplement 1b. [file elife-54051-supp1.docx]

# Supplementary Material: Neural Arbitration between Social and Individual Learning Systems

Andreea O. Diaconescu^1,2,3,4¶^, Madeline Stecy^1,2,5¶^, Lars Kasper^1,2,6^, Christopher J. Burke^2^, Zoltan Nagy^2^, Christoph Mathys^7,8^, Philippe N. Tobler^2^

^1^ Translational Neuromodeling Unit, Institute for Biomedical Engineering, University of Zurich & ETH Zurich, Switzerland;

^2^ Laboratory for Social and Neural Systems Research, Department of Economics, University of Zurich, Switzerland;

^3^ University of Basel, Department of Psychiatry (UPK), Basel, Switzerland;

^4^ Krembil Centre for Neuroinformatics, Centre for Addiction and Mental Health (CAMH), University of Toronto, Canada

^5^ Rutgers Robert Wood Johnson Medical School, New Jersey, United States;

^6^ Institute for Biomedical Engineering, MRI Technology Group, ETH Zürich & University of Zurich, Switzerland;

^7^ Scuola Internazionale Superiore di Studi Avanzati (SISSA), Trieste, Italy;

^8^ Max Planck UCL Centre for Computational Psychiatry and Ageing Research, London, UK

^¶^The authors contributed equally to this work

Correspondence should be addressed to:

Andreea O. Diaconescu, PhD

Krembil Centre for Neuroinformatics (CAMH)

250 College St. M5T 1R8

email: andreea.diaconescu@camh.ca

**Keywords:** hierarchical Bayesian inference, observational learning, reinforcement learning, precision, uncertainty, fMRI, dopamine

## Supplementary Tables

**Supplementary file 1A:**

|  | Hemisphere | x | y | z | *# Voxels* | *F-statistic* |
| --- | --- | --- | --- | --- | --- | --- |
| $\boldsymbol{\varepsilon}_{\boldsymbol{2}}$ card |  |  |  |  |  |  |
| lingual gyrus | R | 10 | -76 | -4 | 10994 | 157.31 |
| inferior occipital gyrus | L | -52 | -74 | 2 | 161 | 40.56 |
|  | R | 52 | -68 | 4 | 466 | 40.32 |
| insula | R | 32 | 22 | -4 | 934 | 53.36 |
|  | L | -34 | 22 | -2 | 745 | 27.62 |
| putamen | L | -30 | 0 | 4 | 331 | 21.44 |
| postcentral gyrus | L | -48 | -22 | 52 | 4230 | 82.43 |
| supramarginal gyrus | R | 58 | -20 | 42 | 1105 | 53.98 |
| dorsolateral PFC | R | 8 | 46 | 30 | 811 | 38.38 |
| substantia nigra | R | 8 | -18 | -14 | 3 | 14.09 |

**Supplementary file 1B:**

|  | Hemisphere | x | y | z | *# Voxels* | *F-statistic* |
| --- | --- | --- | --- | --- | --- | --- |
| $\boldsymbol{\varepsilon}_{\boldsymbol{2}}$ advice |  |  |  |  |  |  |
| cuneus | R | 12 | -88 | 20 | 6082 | 64.33 |
| insula | L | -32 | 20 | -8 | 620 | 42.37 |
|  | R | 32 | 22 | -4 | 663 | 42.18 |
| dorsal middle cingulate gyrus | L | -8 | -16 | 54 | 456 | 37.97 |
| putamen | L | -28 | -4 | 10 | 137 | 37.79 |
| dorsomedial PFC | L | -4 | 22 | 53 | 67 | 24.93 |
| posterior orbitofrontal cortex | L | -28 | 18 | -16 | 459 | 37.19 |
| dorsolateral PFC | R | 40 | 30 | 0 | 159 | 28.80 |
| ventral tegmental area | R | 4 | -16 | -12 | 151 | 49.99 |
|  |  | -4 | -18 | -14 | 150 | 37.94 |
| hippocampus | L | -20 | -28 | -4 | 55 | 33.69 |
