# Supplementary material for: Neural arbitration between social and individual learning systems
Source: eLife. 2020 Aug 11;9:e54051. doi: 10.7554/eLife.54051 (PMC7476763; doi:10.7554/eLife.54051)
Supplement: Transparent reporting form [file elife-54051-transrepform.docx]

***eLife’s* transparent reporting form**

We encourage authors to provide detailed information *within their submission* to facilitate the interpretation and replication of experiments. Authors can upload supporting documentation to indicate the use of appropriate reporting guidelines for health-related research (see [EQUATOR Network](about:blank)), life science research (see the [BioSharing Information Resource](about:blank)), or the [ARRIVE guidelines](about:blank) for reporting work involving animal research. Where applicable, authors should refer to any relevant reporting standards documents in this form.

If you have any questions, please consult our Journal Policies and/or contact us: [editorial@elifesciences.org](about:blank).

**Sample-size estimation**

- You should state whether an appropriate sample size was computed when the study was being designed
- You should state the statistical method of sample size computation and any required assumptions
- If no explicit power analysis was used, you should describe how you decided what sample (replicate) size (number) to use

Please outline where this information can be found within the submission (e.g., sections or figure legends), or explain why this information doesn’t apply to your submission:

We performed an initial power analysis aiming for a minimum power of 0.8 under an effect size of Cohen’s d = 0.42 (as reported in a previous study using a similar experimental task and computational model - Diaconescu et al. 2017). The required sample size for this effect size was N = 35. Assuming a typical drop-out rate of 10%, we recruited N=40 volunteers are included per study.

This information was not initially included in the submission.

**Replicates**

- You should report how often each experiment was performed
- You should include a definition of biological versus technical replication
- The data obtained should be provided and sufficient information should be provided to indicate the number of independent biological and/or technical replicates
- If you encountered any outliers, you should describe how these were handled
- Criteria for exclusion/inclusion of data should be clearly stated
- High-throughput sequence data should be uploaded before submission, with a private link for reviewers provided (these are available from both GEO and ArrayExpress)

Please outline where this information can be found within the submission (e.g., sections or figure legends), or explain why this information doesn’t apply to your submission:

The fMRI experiment has only been conducted once.

The criteria for exclusion of data (trials and subjects) included the following: (i) Incomplete data (as this is a learning task, incomplete data is an exclusion criterion because it prevents one from performing single-trial analysis of the entire session); (ii) Software/Hardware malfunction (e.g., triggers were not sent to the stimulus computer, response box failed); (iii) Participant needed to discontinue before the experiment was complete (e.g., due to fatigue, illness); (iv) Participant pressed the wrong buttons during more than 10% of trials; and (v) Missing parts of the data (e.g., electrode digitization failed, structural MRI could not be recorded, heart rate could not be recorded).

Outlier subjects were included in all analyses.

**Statistical reporting**

- Statistical analysis methods should be described and justified
- Raw data should be presented in figures whenever informative to do so (typically when N per group is less than 10)
- For each experiment, you should identify the statistical tests used, exact values of N, definitions of center, methods of multiple test correction, and dispersion and precision measures (e.g., mean, median, SD, SEM, confidence intervals; and, for the major substantive results, a measure of effect size (e.g., Pearson's r, Cohen's d)
- Report exact p-values wherever possible alongside the summary statistics and 95% confidence intervals. These should be reported for all key questions and not only when the p-value is less than 0.05.

Please outline where this information can be found within the submission (e.g., sections or figure legends), or explain why this information doesn’t apply to your submission:

The statistical tests used are described in the Methods section – see page 38-39, section entitled ‘General Linear Model’. P-values are reported in the figure legends of Figures 6-11.

(For large datasets, or papers with a very large number of statistical tests, you may upload a single table file with tests, Ns, etc., with reference to sections in the manuscript.)

**Group allocation**

- Indicate how samples were allocated into experimental groups (in the case of clinical studies, please specify allocation to treatment method); if randomization was used, please also state if restricted randomization was applied
- Indicate if masking was used during group allocation, data collection and/or data analysis

Please outline where this information can be found within the submission (e.g., sections or figure legends), or explain why this information doesn’t apply to your submission:

Participants were not divided into groups.

No masking was used for data allocation or analysis.

**Additional data files (“source data”)**

- We encourage you to upload relevant additional data files, such as numerical data that are represented as a graph in a figure, or as a summary table
- Where provided, these should be in the most useful format, and they can be uploaded as “Source data” files linked to a main figure or table
- Include model definition files including the full list of parameters used
- Include code used for data analysis (e.g., R, MatLab)
- Avoid stating that data files are “available upon request”

Please indicate the figures or tables for which source data files have been provided:

Source data has been provided for Tables 2a and 2b. The routines for all analyses are available as Matlab code: https://github.com/andreeadiaconescu/arbitration. The instructions for running the code in order to reproduce the results can be found in the ReadMe file. The NII files that produced Figures 6-11 can be reproduced using the scripts available at https://github.com/andreeadiaconescu/arbitration.

**References:**

1. Diaconescu, A.O., Mathys, C., Weber, L.A.E., Kasper, L., Mauer, J., and Stephan, K.E. (2017). Hierarchical prediction errors in midbrain and septum during social learning. Soc. Cogn. Affect. Neurosci. *12*, 618–634.
